# Identification of Mitochondrial Ligands with Hepatoprotective Activity from Notopterygii Rhizoma et Radix Using Affinity Ultrafiltration/Liquid Chromatography/Mass Spectrometry

**DOI:** 10.1155/2019/5729263

**Published:** 2019-12-16

**Authors:** Li Liang, Feng-Jiao Li, Xin Liu, Jian-Kang Mu, Xi Wang, Jin-Cai Dong, Lin-Xi Zeng, Wen Gu, Jing-Ping Li, Xing-Xin Yang, Jie Yu

**Affiliations:** ^1^College of Pharmaceutical Science, Yunnan University of Chinese Medicine, 1076 Yuhua Road, Kunming 650500, Yunnan Province, China; ^2^Beijing Entry-Exit Inspection and Quarantine Bureau, Beijing 100026, China

## Abstract

In recent years, the incidence of diseases associated with hepatic injury has increased in prevalence. Targeting the mitochondria to protect liver function has gained momentum due to their central role in energy production, apoptotic cell death, oxidative stress, calcium homeostasis, and lipid metabolism. In this study, we employed a hepatic mitochondria-based centrifugal ultrafiltration/liquid chromatography/mass spectrometry method (CM-HMC) to identify hepatic mitochondria ligands from medicinal herbs (MHs) including Notopterygii Rhizoma et Radix (NRR) that possess hepatic-protective effects. A total of 4 newly identified mitochondrial ligands were successfully identified by CM-HMC. The mitochondria-regulating activities of 3 of the 4 hits were confirmed using isolated mitochondria. The hepatic-protective effects of one of these hits were validated in carbon tetrachloride-damaged human liver L02 cell models. We have thus identified new natural hepatic-protectants that enhance our understanding of the hepatic-protective mechanisms of MHs. CM-HMC was proven to efficiently screen for mitochondrial ligands from MHs.

## 1. Introduction

The occurrence of liver injury and liver-related morbidity has increased in recent years [1]. Liver damage is mainly caused by external factors, including viruses, alcohol, and drugs, which induce the production of free radicals and cytokines and enhance lipid peroxidation. The culmination of these effects promotes hepatocyte apoptosis and liver fibrosis, resulting in liver cirrhosis and carcinoma [2–4]. Despite tremendous advances in liver drug research, effective and safe hepatoprotective agents remain in urgent demand [5, 6].

Mitochondria play an important role in energy production, apoptotic cell death, oxidative stress, calcium homeostasis, and lipid metabolism [7]. They would be better altering the order of the mitochondria-related functions/consequences. Also, they play a role in the metabolism of amino acids and carbohydrates. Kreb's cycle per se is the “heart” of the metabolism due to their role in connecting several metabolic pathways. A growing body of evidence suggests that mitochondrial disorders result in liver injury through an array of pathways [8–10] including the inhibition of mitochondrial *β*-oxidation and respiratory chain function, collapse of the mitochondrial membrane potential (∆Ψm) through mitochondrial membrane permeability transition protein (mPTP) opening, and damage to the antioxidant defense system. Methods to recover normal mitochondrial function thus represent clinically favorable therapies to cure liver injury.

Medicinal herbs (MHs) can prevent liver disease [11–14]. Whilst it is known that specific MHs remedy mitochondrial dysfunction and thereby promote hepatoprotection [15, 16], the active constituents underpinning these effects remain largely uncharacterized. In our previous studies, an efficient hepatic mitochondria-based centrifugal ultrafiltration/liquid chromatography/mass spectrometry method, which is called capturing method for hepatic mitochondria-targeted compounds (CM-HMC) and is compatible with the search of mitochondrial ligands from complex matrices, was established to expedite the hunt for natural mitochondrial ligands from the extracts of medicinal herbs (MHs) including Peucedani Radix [17]. We confirmed that the method displayed excellent recognition, separation, and identification capabilities, with rapid detection and minimal sample preparation. CM-HMC therefore represents a simple technique that can avoid the isolation of inactive compounds from MH extracts, permitting the direct identification of mitochondrial ligands.

In this study, we initially used CM-HMC to screen for natural mitochondria-targeting compounds from Notopterygii Rhizoma et Radix (NRR, Qianghuo in Chinese). Modern pharmacological research has demonstrated the diaphoretic, antifebrile, antirheumatic, analgesic, antioxidant, antiinflammatory, antiviral, hepatoprotective, and immunosuppressive effects of NRR [18]. Its main chemical constituents are coumarins, phenoloids, and essential oils [19]. In the procedure of CM-HMC analysis, the bioactive molecules that bound to hepatic mitochondria (HM) were isolated by centrifugal ultrafiltration (CU), and isolated fractions were injected into liquid chromatography/mass spectrometry (LC/MS) for rapid isolation and identification. We verified the use of CM-HMC as an efficient method to rapidly screen HM ligands from complex matrices. The results obtained are meaningful to the development of hepatoprotective agents and further our understanding of the protective effects of MHs.

## 2. Experimental Methods

### 2.1. Chemicals and Materials

Notopterol (P1), phellopterin (P2), isoimperatorin (P3), and silybin (SB) were purchased from Chengdu Pufeide Biological Technology Co., Ltd. (Chengdu, China). Rhodamine 123 (Rh123) was obtained from Dalian Meilun Biotechnology Co., Ltd. (Dalian, China). Cyclosporine A (CsA) was provided by J&K Scientific Ltd. (Beijing, China). Pioglitazone hydrochloride (PH) was purchased from Ark Pharm Inc. (Chicago, IL, USA). Dulbecco's Modified Eagle's Medium (DMEM) and the Bicinchoninic Acid (BCA) Protein Determination Kit were provided by Nanjing Built Biological Engineering Research Institute (Nanjing, China). Mitochondria Separation Kit was supplied by Genmed Scientifics Inc. (Arlinghton, MA, USA). Fetal bovine serum (FBS) was purchased from Gibco (Grand Island, NY, USA). HPLC-grade methanol, acetonitrile and formic acid were obtained from Merck (Darmstadt, Germany). Deionized water was supplied by a Milli-Q System (Millipore, Bedford, MA, USA). All other reagents were of analytical grade or higher. NRR (which originates from the dried rhizome and root of *Notopterygium incisum* Ting ex H. T. Chang and *Notopterygium franchetii* H. de Boiss.) was purchased from Lvsheng Business Department of Medicinal Materials (purchased on the 16^th^ of June, 2016, Kunming, China). Samples were authenticated by Professor Jie Yu, and NRR specimens (No. 7586) were deposited in the Key Laboratory of Preventing Metabolic Diseases of Traditional Chinese Medicine, Yunnan University of Traditional Chinese Medicine (Kunming, China).

### 2.2. Animals

Healthy male Sprague–Dawley (SD) rats (200 ± 50 g) were obtained from Liaoning Changsheng Biotechnology Co., Ltd. (Liaoning, China). All animals were adjusted to a controlled environment (22 ± 1°C; 60 ± 10% humidity; and a 12 h/12 h light/dark cycle) with free access to water and commercial laboratory complete food throughout the study period. All procedures involving animals complied with the Guide for the Care and Use of Laboratory Animals published by the US National Institutes of Health and approved by the Biomedical Ethical Committee of Yunnan University of Chinese Medicine (R-0620160026). All reasonable efforts were made to minimize the animals' suffering.

### 2.3. Preparation of Analytical Solutions

Working solutions of NRR (495 mg/mL) were prepared by dissolving the lyophilized powder of the NRR extract (see supplementary information for the preparation procedure) in DMSO. For *in vitro* pharmacological assessment, analytical solutions of the standard substances derived from NRR extract (P1, P2, and P3) and used as pharmacological experimental tools (SB, CsA, and PH) were prepared by dissolution in DMSO and dilution in physiological saline to the appropriate concentrations. All solutions were stored at 4°C in the dark.

### 2.4. Preparation of HM Suspension

HM suspension was prepared from rat liver using Mitochondria Isolation Kit (which permits organelle release followed by differential centrifugation) as previously described [17]. Briefly, livers isolated from SD rats were rapidly placed into ice-cold Reagent A to remove the blood, sliced into 1 mm^3^ sections, and homogenized using a Dounce glass homogenizer (Kimble/Kontes, Vineland, NJ, USA) in working solution (3.2 mL Reagent B plus 0.8 mL Reagent C and 16 *μ*L Reagent F). Following centrifugation at 1,000 ×*g* for 10 min, supernatants were collected and centrifuged at 10,000 ×*g* for 10 min to obtain the HM pellet. Pellets were resuspended in Reagent E to a concentration of 1.0 g/L. Mitochondrial protein concentrations were determined using BCA assays. A sample of the HM suspension was heated for 1 h in boiling water bath for the production of a denatured control sample with no biological functions ([Supplementary-material supplementary-material-1]).

### 2.5. CM-HMC Screening

The screening of CM-HMC was based on previously reported methods [17]. Briefly, 5 *μ*L of NRR was incubated with 200 *μ*L of HM suspension for 90 min at 37°C and filtered through a 10 kDa molecular weight cutoff ultrafiltration membrane (0.5 mL; Microcon YM-10, Millipore Co., Bedford, MA, USA) by centrifugation at 14,000 ×*g* for 25 min at 4°C. Captured pellets were washed three times in 200 *μ*L of ammonium acetate buffer (50 mM, pH 7.5) by centrifugation at 14,000 ×*g* for 25 min at 4°C. Bound ligands were released from the HM by sonication in 400 *μ*L of 80% aqueous methanol for 20 min, followed by centrifugation at 14,000 ×*g* for 25 min at room temperature. Ultrafiltrates were dried under a stream of nitrogen and reconstituted in 100 *μ*L of 80% methanol. Samples containing the screened molecules were analyzed by LC/MS. Control tests for nonspecific binding were performed using denatured HM. LC/MS peaks that enhanced significantly in area in the experiments comparing to the control containing denatured HM (percent of difference of peak areas between experiment and control (Δ*P*) was higher than 20%) indicated specific binding and were deemed as the mitochondrial ligands, which was confirmed in our previous studies [17, 20, 21]. All screening tests were independently performed in triplicate and analyzed in duplicate. The ∆*P* values were calculated using the following formula:

(1) $$\begin{array}{c} P=\frac{\left(P_{\text{e}}-P_{\text{c}}\right)}{P_{\text{e}}}\times 100, \end{array}$$

where *P*_e_ and *P*_c_ are the peak areas in experiment and control, respectively.

### 2.6. Determination of mPTP Opening, ∆Ψm, and ATPase Activity in Isolated HM

mPTP opening was measured through the Ca^2+^-induced swelling of isolated HM as previously described [17, 20, 21]. Mitochondrial swelling induced by mPTP opening is measured by the reduction of absorbances at 520 nm (*A*_520_). Briefly, isolated HM were resuspended in swelling buffer (120 mM KCl, 20 mM MOPS, 10 mM Tris-HCl and 5 mM KH_2_PO_4_, pH 7.4) to a final concentration of 0.25 g/L and preincubated with test compounds for 3 min at room temperature prior to the addition of 250 *μ*M CaCl_2_ to induce mPTP opening for 15 min. Changes in *A*_520_ were measured in triplicate using a UV-VIS spectrophotometer (AOE instrument (Shanghai) Co., Ltd., Shanghai, China).

∆Ψm was determined through Rh123 fluorescence quenching as previously described [22]. At high ΔΨm levels, the majority of Rh123 aggregated in the mitochondrial matrix and was quenched. At lower ΔΨm levels, Rh123 was released, inducing Rh123 fluorescence dequenching [22]. Briefly, isolated HM (1 g/L) were resuspended in measurement medium (0.25 M sucrose, 5 mM MgCl_2_, 10 mM KCl, 5 mM KH_2_PO_4_, 10 mM HEPES, and 10 mM succinate, pH 7.4) and incubated with the assayed compounds and Rh123 (5 *μ*L; 1 g/L) for 30 min at 37°C. PH (880 *μ*M) was then added to induce Rh123 fluorescence quenching for 15 min. Changes in Rh123 fluorescence were determined in triplicate on an Infinite M200 Pro Multifunctional Microplate Reader (Dongsheng Innovation Biotechnology Co., Ltd., Beijing, China) at 484 nm excitation and 534 nm emission.

ATPase activity was measured through the PH-induced injury of isolated HM [22]. Isolated HM (1 g/L) were resuspended in measurement medium and incubated with the test compounds for 20 min at 37°C prior to the addition of 880 *μ*M PH for 20 min to inhibit ATPase activity. Changes in Na^+^-K^+^-ATPase and Ca^2+^-Mg^2+^-ATPase activity were measured on a SpectraMax Plus 384 Microplate Reader (Molecular Devices, Sunnyvale, CA, USA) using an ATPase Determination Kit (Nanjing Built Biological Engineering Research Institute, Nanjing, China) according to the manufacturer's instructions.

### 2.7. In Vitro Hepatoprotective Assessment

A hepatocyte model of CCl_4_-induced injury was employed to evaluate the hepatoprotective activity of the hit compounds as previously described [22]. L02 cells (1 × 10^5^ cells per well) were plated into 96-wells and cultured in DMEM plus 10% FBS at 37°C in a humidified atmosphere of 5% CO_2_. After attachment, cells were treated with each test compound for 18 h and hepatocellular injury was induced through the addition of 10 *μ*M CCl_4_ for 6 h. Cell viability (CCK-8 assay), malondialdehyde (MDA) levels, and alanine transaminase (ALT), aspartate transaminase (AST), and superoxide dismutase (SOD) activity were assessed in a SpectraMax Plus 384 Microplate Reader (Molecular Devices, Sunnyvale, CA, USA) using commercially available kits (Nanjing Built Biological Engineering Research Institute, Nanjing, China) according to the manufacturer's instructions.

### 2.8. Data Analyses

Data are presented as mean ± S.D. A one-way analysis of variance (ANOVA, Dunnett's method) was performed for statistical comparisons between the treatment groups. Data analyses were performed using SPSS software (13.0 for Windows; SPSS, Chicago, IL, USA). *P* < 0.05 (two-tailed) was considered significant.

## 3. Results and Discussion

### 3.1. CM-HMC

CM-HMC was developed through the coupling of CU with LC/MS to directly identify HM-targeted constituents from complex MH samples [17]. Using CM-HMC, bioactive constituents against specific targets in HM could be isolated. Inactive impurities were excluded using CU. The chemical structures of the corresponding compounds were assigned by LC/MS. CM-HMC permitted the rapid recognition, isolation, and identification of HM-targeted compounds that were richly validated in positive and negative control conditions [17]. CM-HMC therefore represented an efficient method to rapidly identify HM-targeted chemicals with potential therapeutic benefits for mitochondrial disorders from MHs.

### 3.2. Effects of the Screening Conditions

CM-HMC was applied for the capture of natural mitochondrial ligands from NRR extract. To optimize its performance and further understand its major influencing factors, the effects of HM concentration, sample concentration, and incubation time were investigated.

The HM concentration dictates the sensitivity of CM-HMC. Higher HM concentration provides enhanced sensitivity, permitting compound identification from MHs [17, 20, 21]. In this study, HM concentrations of 0.25, 0.50, and 1.00 g/L were assessed for the screening of bioactive constituents from NRR extract. The number of identified active molecules significantly increased from 0.25 to 0.5 g/L ([Supplementary-material supplementary-material-1]) but was unchanged between 0.50 and 1.00 g/L. Thus, 0.50 g/L was deemed optimal.

Sample concentration can influence the identification of active chemicals. The constituents in MHs of low concentrations cannot be easily identified by CM-HMC, because they may not be detected by LC/MS or may be easily replaced competitively by higher-content constituents. Conversely, excessive sample concentration can compromise sample purity and increase the likelihood of false positives. In this study, NRR concentrations of 3.10, 6.19, and 12.38 g/L were independently assessed. The number of active compounds increased with sample concentration ([Supplementary-material supplementary-material-1]), with 12.38 g/L identified as optimal. In our previous studies, 2.500, 7.500, 10.50, and 15.50 g/L permitted the identification of mitochondrial ligands from other MHs [20, 21]. The optimal sample concentration therefore varies according to the chemical constituents of each individual MH and should be independently optimized prior to CM-HMC screening.

The incubation time can also influence sample isolation. Short incubations can miss active molecules that bind to HM, whilst extended incubations may induce structural changes in the bound chemicals, leading to both false positive and negative results. In this study, different incubation times (60, 90, and 120 min) were assessed. The results suggested that 90 min was sufficient for screening the NRR extract ([Supplementary-material supplementary-material-1]), with 60 or 90 min representing the optimal incubation time for other MHs [17, 20, 21]. The optimal incubation time for screening mitochondrial ligands from MHs therefore ranges from 60 to 90 min, which should be optimized for each individual MH to achieve optimal screening performance.

### 3.3. Identification of HM-Targeting Molecules from NRR Extracts Using CM-HMC

**Figure 1:**
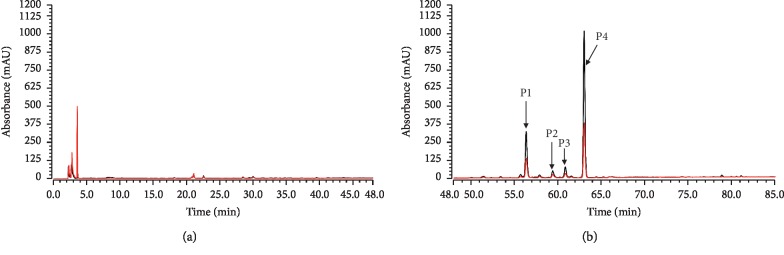
Identification of HM-targeting molecules from NRR extract using CM-HMC. Compared to denatured HM (red line), HPLC chromatograms of NRR extract, (a) 0–47 min and (b) 48–85 min, revealed 4 peaks (P1–P4) due to specific HM binding. Analysis conditions of the NRR extract are shown in the supplementary information.

**Figure 2:**
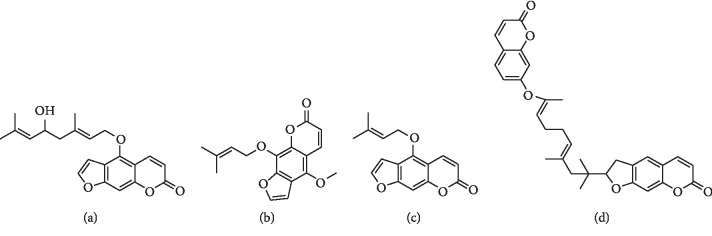
Chemical structures of the assigned HM-targeting compounds obtained from the NRR extract. (a) P1. (b) P2. (c) P3. (d) P4.

CM-HMC was used to identify HM-targeting molecules from NRR extract.  Figure 1 shows the chromatogram of the NRR sample analyzed by CM-HMC in which 4 distinct peaks were identified compared to denatured control samples (Δ*P* > 20%, Table 1), indicating specific HM binding. The chemical structures of the 4 peaks were assigned as coumarin (Figure 2), through analyzing the UV, MS, and MS^*n*^ information provided by LC/MS (Table 1) and through comparison with previously reported data and standards [23, 24]. It is essential to indicate that several ingredients with a low peak area in the NRR extract (Figure 1) that showed area enhancement may have activity, but should not be assigned as HM ligands, considering they were unrepeatedly captured by CM-HMC. This may be attributed to the low abundance in the sample, low response to LC/MS, and/or weak binding with HM.

These results further indicated that CM-HMC can identify HM-targeted chemicals from MHs extracts. Due to the negative membrane potential of HM, positively charged molecules can spontaneously accumulate in HM without specific ligand-target interactions [20]. Thus, HM can bind active molecules through affinity-based and electrostatic interactions. The recognized fractions were rapidly isolated using the CU technique and separated fractions were subjected to LC/MS analysis.

### 3.4. Effects of Hits on mPTP Opening, ∆Ψm, and ATPase Activity in Isolated HM

**Figure 3:**
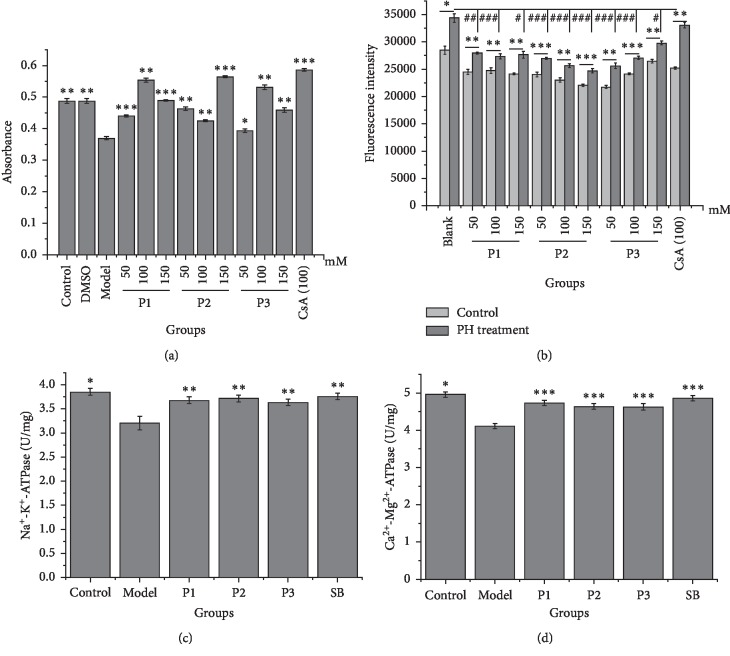
Effects of the HM-targeting compounds on mPTP opening (a), ∆Ψm (b), Na^+^-K^+^-ATPase activity (c), and Ca^2+^-Mg^2+^-ATPase activity (d) in isolated HM (*n* = 5). ^*∗*^*P* < 0.05, ^*∗∗*^*P* < 0.01, and ^*∗∗∗*^*P* < 0.001 compared to the model control group (a, c, d) or compared to control and PH treatment (b), determined under identical conditions. ^##^*P* < 0.05, ^###^*P* < 0.001 compared to PH treatment. P1, notopterol; P2, phellopterin; P3, isoimperatorin; CsA, cyclosporin A; SB, silybin; and PH, pioglitazone hydrochloride.

To validate the ability of the identified compounds to bind to HM, we assessed their effects on HM functionality, including mPTP opening, ∆Ψm, and ATPase activity. As shown in Figure 3(a), 250 *μ*M CaCl_2_ (known to open mPTP [20]) led to a reduction in *A*_520_ of HM suspensions that could be prevented by P1, P2, P3. CsA (a known inhibitor of mPTP opening) similarly prevented the reduction in *A*_520_ confirming that this effect was attributed to mPTP opening.

As shown in Figure 3(b), the fluorescence intensity of the HM suspensions notably increased following PH treatment, suggesting a reduction in the ∆Ψm of the HM [25]. When HM preparations were preincubated with P1, P2, and P3 for 30 min, the PH-induced fluorescence changes were notably inhibited, indicating that the compounds prevent the PH-induced decrease of ∆Ψm.

As shown in Figures 3(c) and 3(d), the Na^+^-K^+^-ATPase and Ca^2+^-Mg^2+^-ATPase activity of the HM suspensions significantly decreased following PH treatment due to a loss of ATPase activity [25]. When HM were preincubated with P1, P2, and P3 for 20 min, the loss of ATPase activity induced by PH was inhibited to levels comparable to positive control samples (SB). These data suggested that the test hits were the potential mitochondrial ligands that can directly recover HM functionality, indicating credible results captured by CM-HMC. Combining with the results obtained from CM-HMC, the other one hit was the potential mitochondrial ligands regulating HM functions, which merit further investigation. Additionally, the specific targets that these mitochondrial ligands may bind are valuable to be indentified in future research.

### 3.5. In Vitro Hepatoprotective Activities of Hits

**Figure 4:**
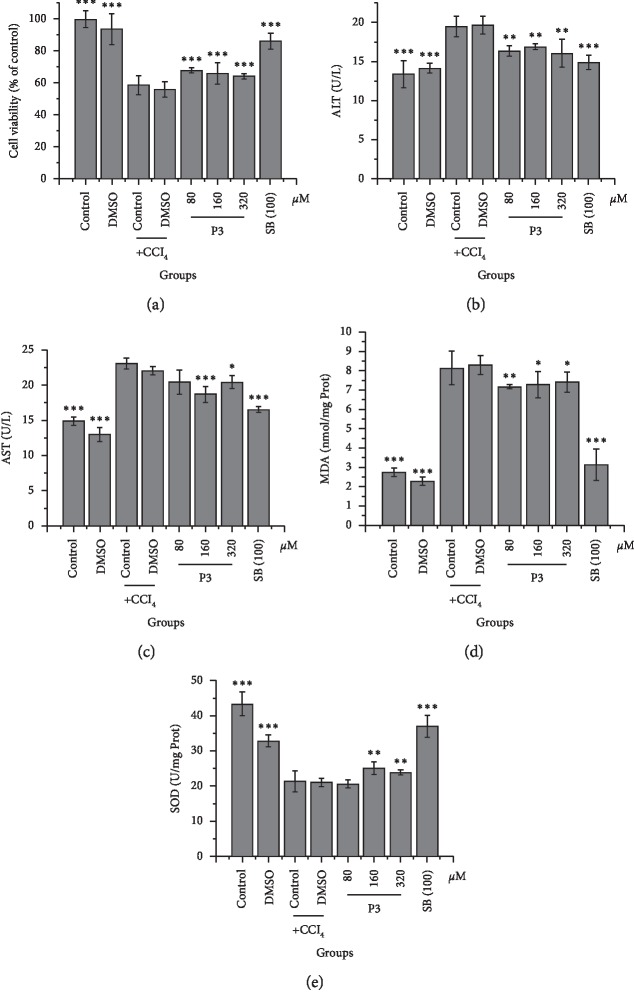
Effects of the tested compounds on CCl_4_-induced hepatocyte injury (*n* = 5). ^*∗*^*P* < 0.05, ^*∗∗*^*P* < 0.01, and ^*∗∗∗*^*P* < 0.001 compared to the model control group under identical conditions. P3, isoimperatorin; SB, silybin.

The hepatoprotective activities of the compounds were initially evaluated in hepatocyte models of CCl_4_-induced injury. As shown in Figure 4, both compounds P3 and SB (a known hepatoprotective compound [26]) significantly inhibited the detrimental effects of CCl_4_-induced hepatocyte injury, including the loss in viability and SOD and increased ALT, AST, and MDA level. Similarly, the ability of compound P2 to inhibit CCl_4_-induced hepatotoxicity has been reported [27]. The effects of P1 and P4 on hepatocellular protection warrants further investigation.

In summary, CM-HMC coupled to the measurement of HM indexes indicated that the hit compounds acted on HM to influence HM functions and further perform their hepatoprotective abilities, which is helpful for natural hepatic-protectants development from MHs and interpretation of therapeutic principles of MHs.

## 4. Conclusion

In this study, we screened HM-targeting molecules from NRR using CM-HMC. Four HM ligands were successfully identified. The direct HM-regulating activities of three hit molecules were confirmed in mPTP opening, ∆Ψm, and ATPase assays. Moreover, the hepatoprotective activities of P3 were highlighted in cell-based systems of hepatocyte damage. The results highlight the benefit for HM-targeting hepatic-protectants development from MHs and in-depth elucidation of hepatic-protective mechanisms of MHs. CM-HMC was further confirmed to be a proposal for the efficient discovery of HM-targeted hepatic-protectants from MHs.

## Figures and Tables

**Table 1:** LC/MS data and assignment of the four bioactive constituents in NRR extract.

NO.	*t*_R_ (min)	∆*P*^b^ (%, *n* = 3)	UV *λ*max (nm)	[M + H]^+^ [M + Na]^+^*m*/*z*	ESI-MS^*n*^ (+) *m*/*z* (abundance)	[M − H]^+^*m*/*z*	ESI-MS^*n*^ (-) *m*/*z* (abundance)	Predicted formula	Meas (*m*/*z*)	Pred (*m*/*z*)	Diff (ppm)	DBE	Assigned identification
^a^P1	56.414	30.1 ± 3.5	250, 310	355.1537	—	353.1408	MS^2^ (353): 353 (100), 254 (26), 297 (18), 210 (13), 214 (12), 269 (10)	C_21_H_22_0_5_	353.1408	353.1358	4.67	11	Notopterol
^a^P2	59.285	37.4 ± 4.2	295	301.1074	MS^2^ (301): 233 (100)	299.2598	MS^2^ (299): 229 (100), 69 (43)	C_17_H_16_0_5_	301.1074	301.1414	1.16	9	Phellopterin
^a^P3	60.857	28.3 ± 2.6	220, 250, 310	271.0969	MS^2^ (271): 203 (100), MS^3^ (203): 147 (73), 119 (38), 175 (10)	269.2127	MS^2^ (269): 62 (100), 254 (49), 201 (29), 69 (26), 238 (16), 221 (15)	C_16_H_14_0_4_	271.0969	271.0967	1.29	10	Isoimperatorin
P4	63.017	42.4 ± 5.1	230, 330	299.1102	MS^2^ (299): 175 (100), 231 (12)	297.2427	—	C_19_H_22_0_3_	298.1611	298.1867	4.65	9	7-Geranyloxycoumarin

^a^Comparison to standards. ^b^Δ*P* was calculated using Δ*P* = (*P*_*e*_ – *P*_*c*_)/*P*_*e*_ × 100, where *P*_*e*_ and *P*_*c*_ are the peak areas in the experimental and control groups, respectively. Data were obtained from 3 independent experiments and are presented as the mean ± SD.

## Data Availability

The data used to support the findings of this study are available from the corresponding author upon request.

## Supplementary Materials

Supplementary Materials The supplementary experimental procedures comprise the preparation of lyophilized NRR extract and analytical condition of LC/MS. Figure S1: changes in the mitochondrial membrane potential in isolated hepatic mitochondria after heating in boiling water for 1 h; Figure S2: effects of mitochondrial concentration on the screening of bioactive constituents from NRR extract; Figure S3: effects of NRR sample concentration on the screening of mitochondrial ligands; Figure S4: effects of incubation time on the screening of bioactive compounds from NRR extract.
