# Supplementary material for: Identification of Mitochondrial Ligands with Hepatoprotective Activity from Notopterygii Rhizoma et Radix Using Affinity Ultrafiltration/Liquid Chromatography/Mass Spectrometry
Source: Biomed Res Int. 2019 Dec 16;2019:5729263. doi: 10.1155/2019/5729263 (PMC6948297; doi:10.1155/2019/5729263)
Supplement: Supplementary Materials — The supplementary experimental procedures comprise the preparation of lyophilized NRR extract and analytical condition of LC/MS. Figure S1: changes in the mitochondrial membrane potential in isolated hepatic mitochondria after heating in boiling water for 1 h; Figure S2: effects of mitochondrial concentration on the screening of bioactive constituents from NRR extract; Figure S3: effects of NRR sample concentration on the screening of mitochondrial ligands; Figure S4: effects of incubation time on the screening of bioactive compounds from NRR extract. [file 5729263.f1.doc]

**SUPPLEMENTARY INFORMATION**

**Identification of Mitochondrial ligands with Hepatoprotective Activity from Notopterygii Rhizoma et Radix using Affinity Ultrafiltration/Liquid Chromatography/Mass Spectrometry**

Li Liang ^a,#^, Feng-Jiao Li ^a,#^, Xin Liu ^b^, Jian-Kang Mu ^a^, Xi Wang ^a^, Jin-Cai Dong ^a^, Lin-Xi Zeng ^a^, Wen Gu ^a^, Jing-Ping Li ^a^, Xing-Xin Yang ^a^,*, Jie Yu ^a^,*

^a^ College of Pharmaceutical Science, Yunnan University of Chinese Medicine, 1076 Yuhua Road, Kunming 650500, Yunnan Province, P.R. China

^b^ Beijing Entry-Exit Inspection and Quarantine Bureau, Beijing 100026, China

^#^ Both authors contributed equally to this work

**Corresponding author:**

Dr. Xing-Xin Yang (Tel./Fax.: +86-871-65933303; Email: [yxx78945@163.com](mailto:cz.yujie@gmail.com)); Dr. Jie Yu (Tel./Fax.: +86-871-65933303; Email: [cz.yujie@gmail.com](mailto:cz.yujie@gmail.com))

**Table of Contents**

**Supplementary Experimental Procedures**

**Supplementary Figures and Table**

1. **Fig. S1.** Changes in the mitochondrial membrane potential in isolated hepatic mitochondria after heating in boiling water for 1 h.
2. **Fig. S2.** Effects of mitochondrial concentration on the screening of bioactive constituents from NRR extract.
3. **Fig. S3.** Effects of NRR sample concentration on the screening of mitochondrial ligands.
4. **Fig. S4.** Effects of incubation time on the screening of bioactive compounds from NRR extract.

**Supplementary Experimental Procedures**

**Preparation of lyophilized Notopterygii Rhizomaet Radix (NRR) extract**

Pulverized dried NRR samples (50 g) were immersed in methanol (500 mL) for 30min, and ultrasonicated (500 W, 40 KHz) for 60 min. Extracted solutions were filtered, and the residues ultrasonicated (500 W, 40 KHz) in 70% methanol (400 mL) for an additional 60 min. The filtrates were collected after leaching. Then, the two filtrates were mixed and concentrated with an N-1100D-WDrotatory evaporator (Ai Lang Instrument Co., Ltd., Shanghai, China) at 45 ˚C under reduced pressure. Finally, the concentrate was lyophilized using a FD8-10B freeze dryer (SIM International Group Co. Ltd., Newark, DE, USA).The lyophilized powder of the NRR extract was stored in the dryer at room temperature until use.

**Analytical condition of LC/MS**

LC/MS analyses were performed on an UHPLC Dionex Ultimate 3000 system connected to a Thermo Scientiﬁc Q-ExactiveTM hybrid quadrupole-orbitrap mass spectrometer with heated-electrospray ionization (HESI) probe (Thermo Fisher Scientiﬁc, San Jose, CA, USA). The UHPLC system was comprised of a quaternary pump, a column box, a PDA detector and an autosampler with temperature control function.

The UHPLC-PDA conditions were as follows: 1) column: Agilent Zobax SB-C18 column (250 mm × 4.6 mm I.D., 5 μm); 2) mobile phase: water (A) and acetonitrile (B) with a gradient program (0-10 min, 5%B→15%B; 10-20 min, 15%B→25%B; 20-30 min, 25%B→35%B; 30-45 min, 35%B→45%B; 45-60 min, 45%B→65%B; 60-70 min, 65%B→80%B; 70-80 min, 80%B→95%B; 80-85 min, 95%B→100%B); 3) sample injection volume: 20 μL; 4) column temperature: 30 °C; 5) flow rate: 1.0 mL/min; 6) UV and visible light spectra were obtained by scanning from 200 nm to 700 nm; 7) HPLC chromatograms were recorded at 203 nm.

The HESI-MS*^n^* parameters were as follows: 1) detection mode: positive and negative ion; 2) flow rate: 0.2 mL/min (split from HPLC effluent); 3) heat block and curved desolvation line temperature: 250 °C; nebulizing nitrogen gas flow: 1.5 L/min; Interface voltage: (+) 3.5 kV, (-) -2.8 kV; 4) mass range: MS, m/z 100~1000; MS^2^ and MS^3^, m/z 50~1000; 5) dynamic exclusion time: 10 s; 6) Workstation: Xcalibar 3.0.63 for LC coupled with data processing, molecular prediction and precise molecular weight calculations.

**Supplementary Figures and Tables**

**

**

**Fig. S1.** Change in the mitochondrial membrane potential in isolated hepatic mitochondria after heating in boiling water for 1 h. Mitochondrial membrane potentials were assessed as the difference in rhodamine 123 uptake by normal and heated mitochondria and expressed in fluorescence intensity units. Values are presented as the mean of three independent experiments (S.D.). Statistical significance between normal and heated mitochondria is marked by * (*P*< 0.05).


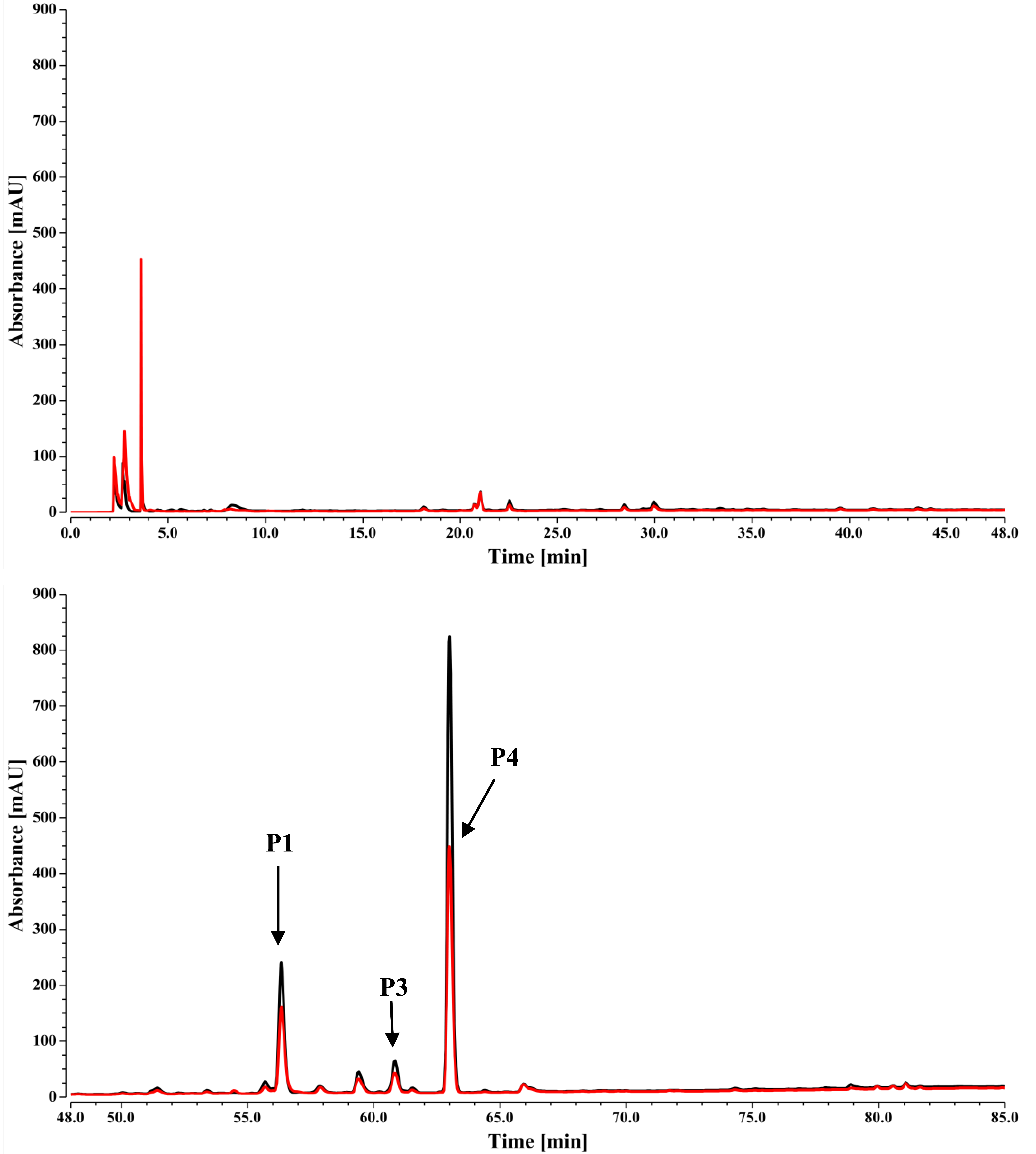

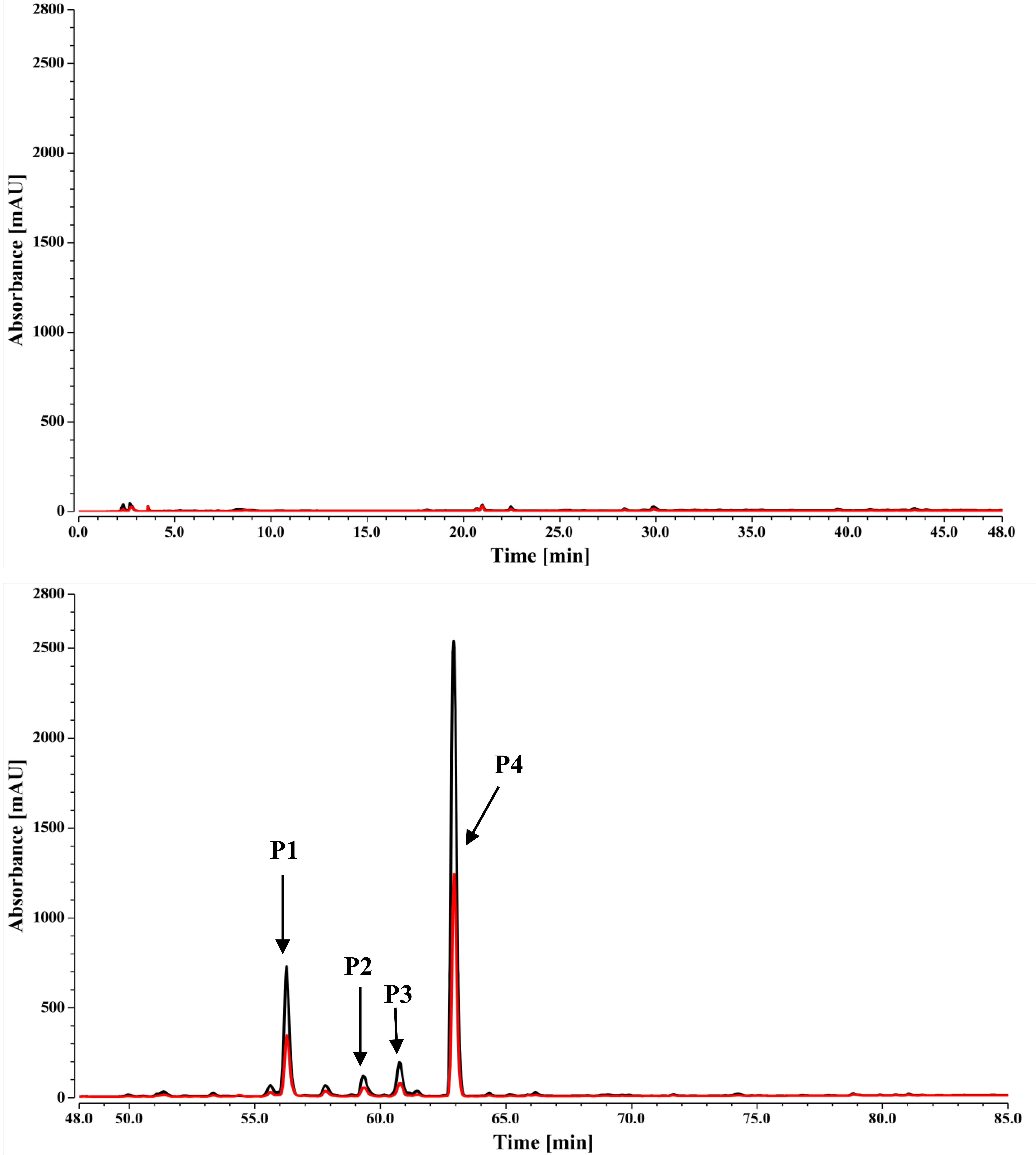

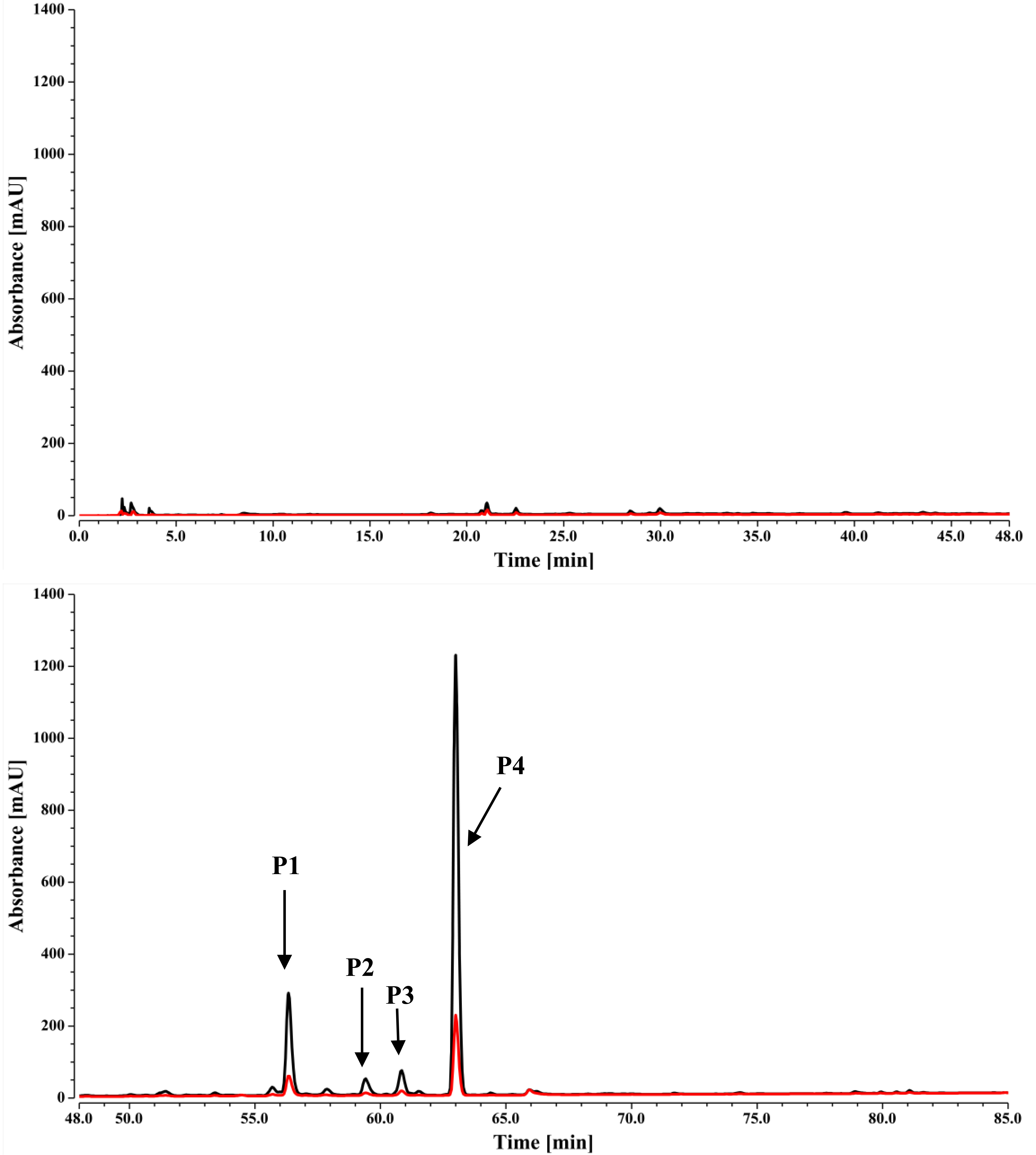


**(A1)**

**(A2)**

**(B1)**

**(B2)**

**(C1)**

**(C2)**

**Fig.S2.** Effects of mitochondrial concentration on the screening of bioactive constituents from NRR extract. Concentrations of mitochondria were 0.25 (A1 and A2; A1, 0–48 min; A2, 48–85 min), 0.50 (B1 and B2; B1, 0–48 min; B2, 48–85 min) and 1.00 g/L (C1 and C2; C1, 0–48 min; C2, 48–85 min). Compared to controls, i.e., denatured hepatic mitochondria (red line), HPLC chromatograms of analyzed NRR sample exhibited four peaks (P1–P4) that were enhanced because of specific binding to hepatic mitochondria (black line). The concentration of the NRR sample and incubation time were 12.38 g/L and 90 min, respectively.


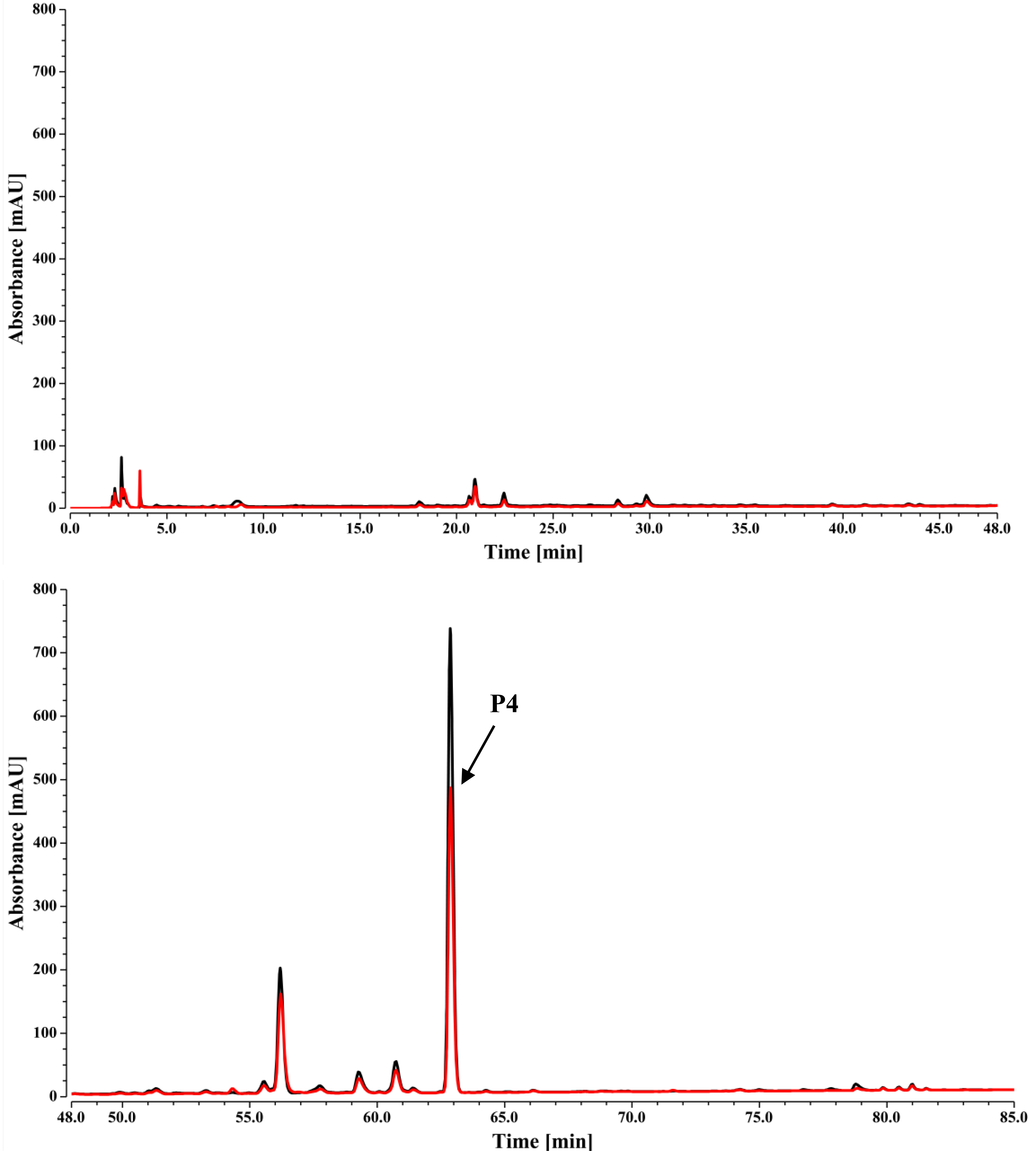


**(A1)**

**(A2)**


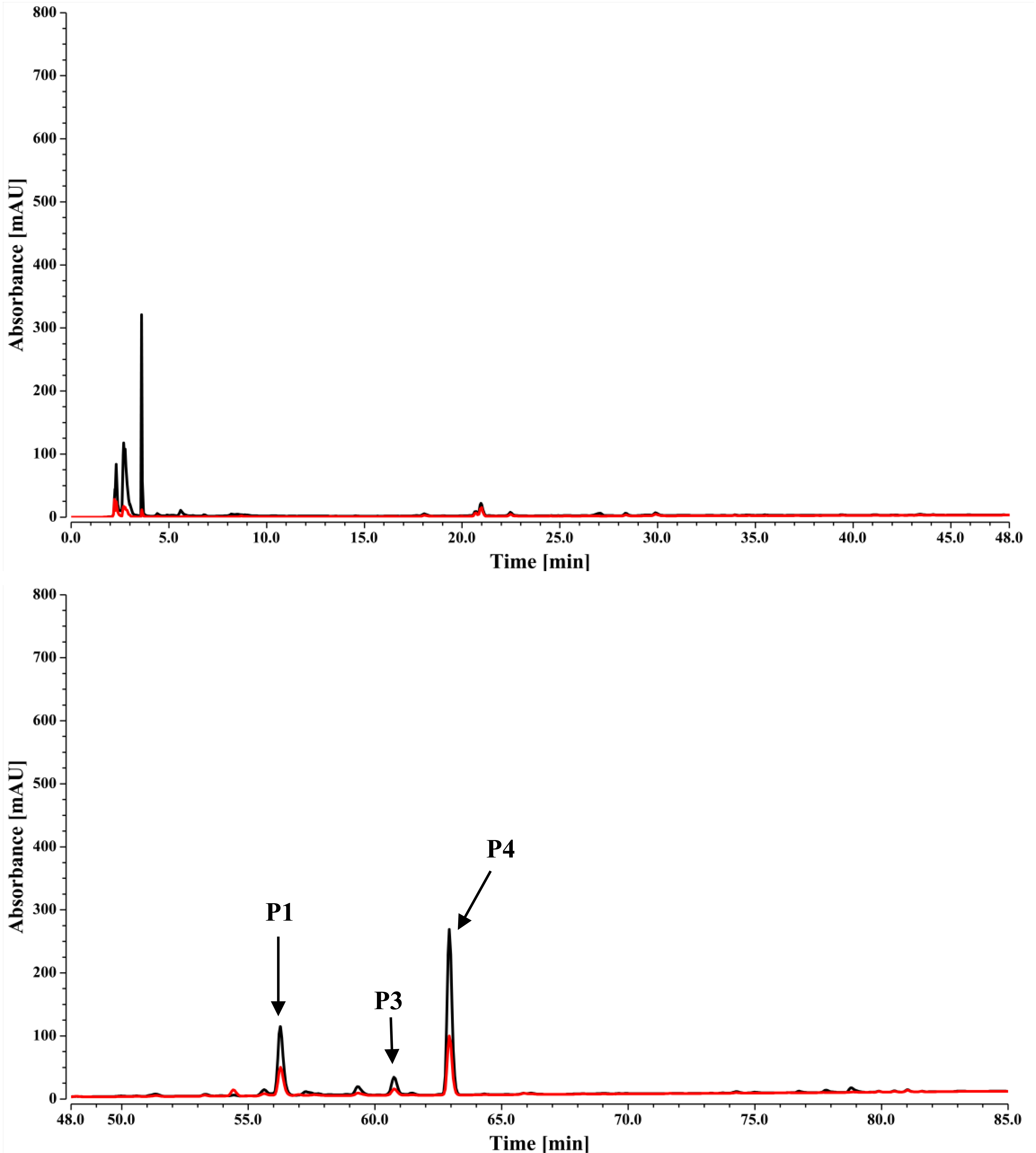


**(B1)**

**(B2)**


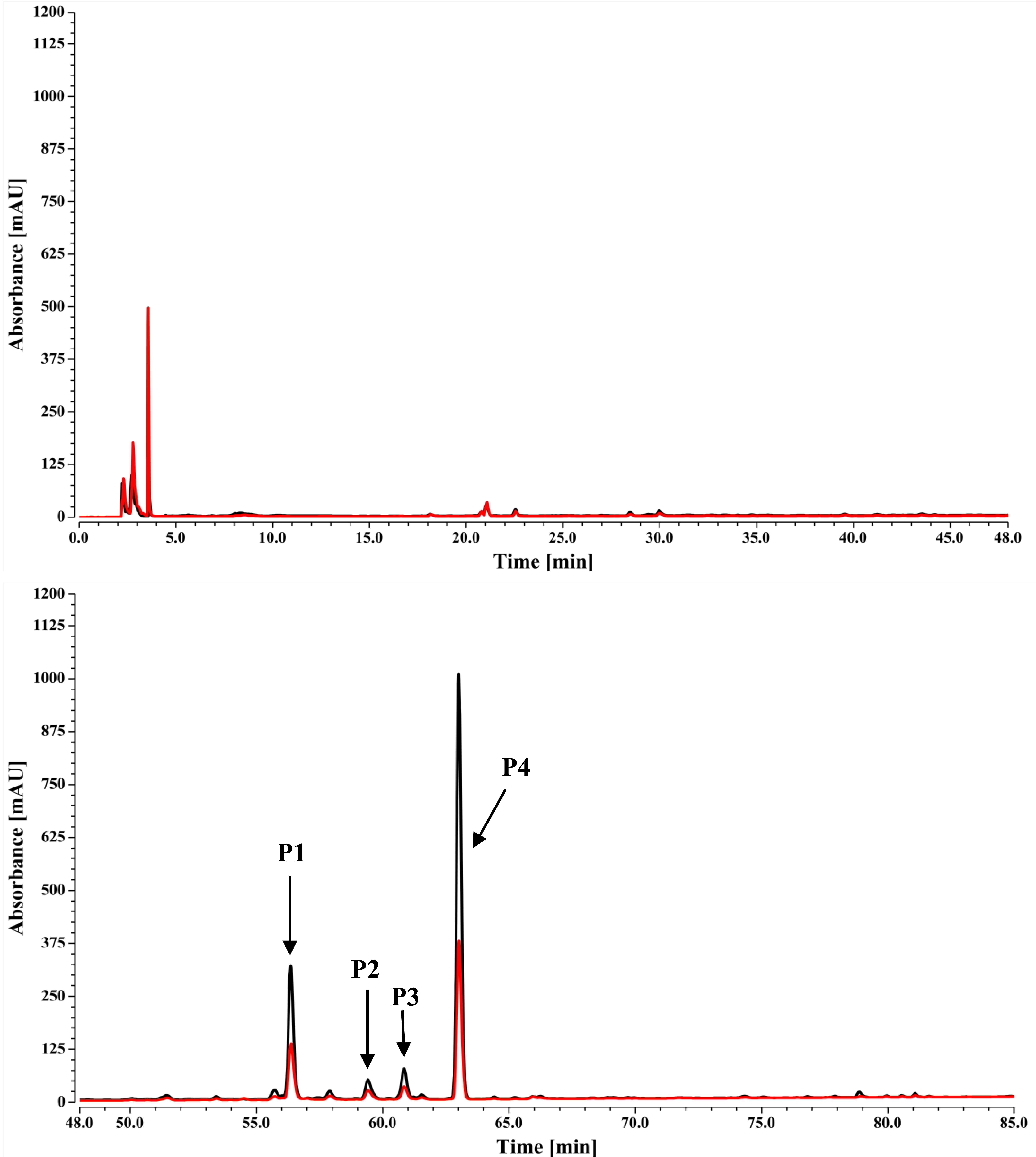


**(C1)**

**(C2)**

**Fig. S3.** Effects of NRR sample concentration on the screening of mitochondrial ligands. The concentrations of NRR sample were 3.10 (A1 and A2; A1, 0–48 min; A2, 48–85 min), 6.19 (B1 and B2; B1, 0–48 min; B2, 48–85 min) and 12.38 g/L (C1 and C2; C1, 0–48 min; C2, 48–85 min). HPLC chromatograms of NRR sample are displayed for the ultrafiltrates obtained from active (black line) and denatured mitochondria (red line). Four peaks (P1–P4) showed significant area enhancement compared to the controls. The concentration of hepatic mitochondria and incubation time were 0.50 g/L and 90 min, respectively.


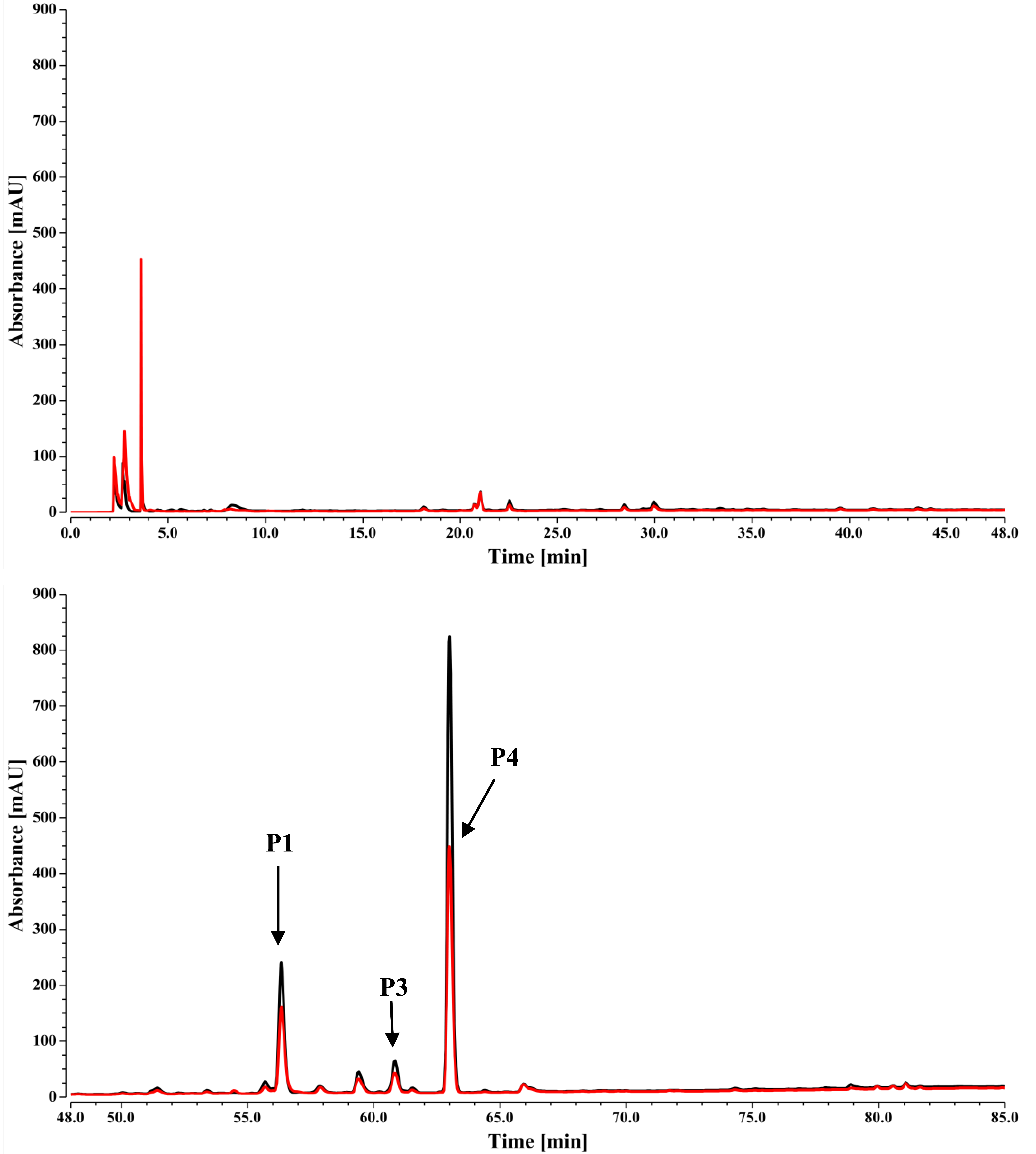


**(A1)**

**(A2)**


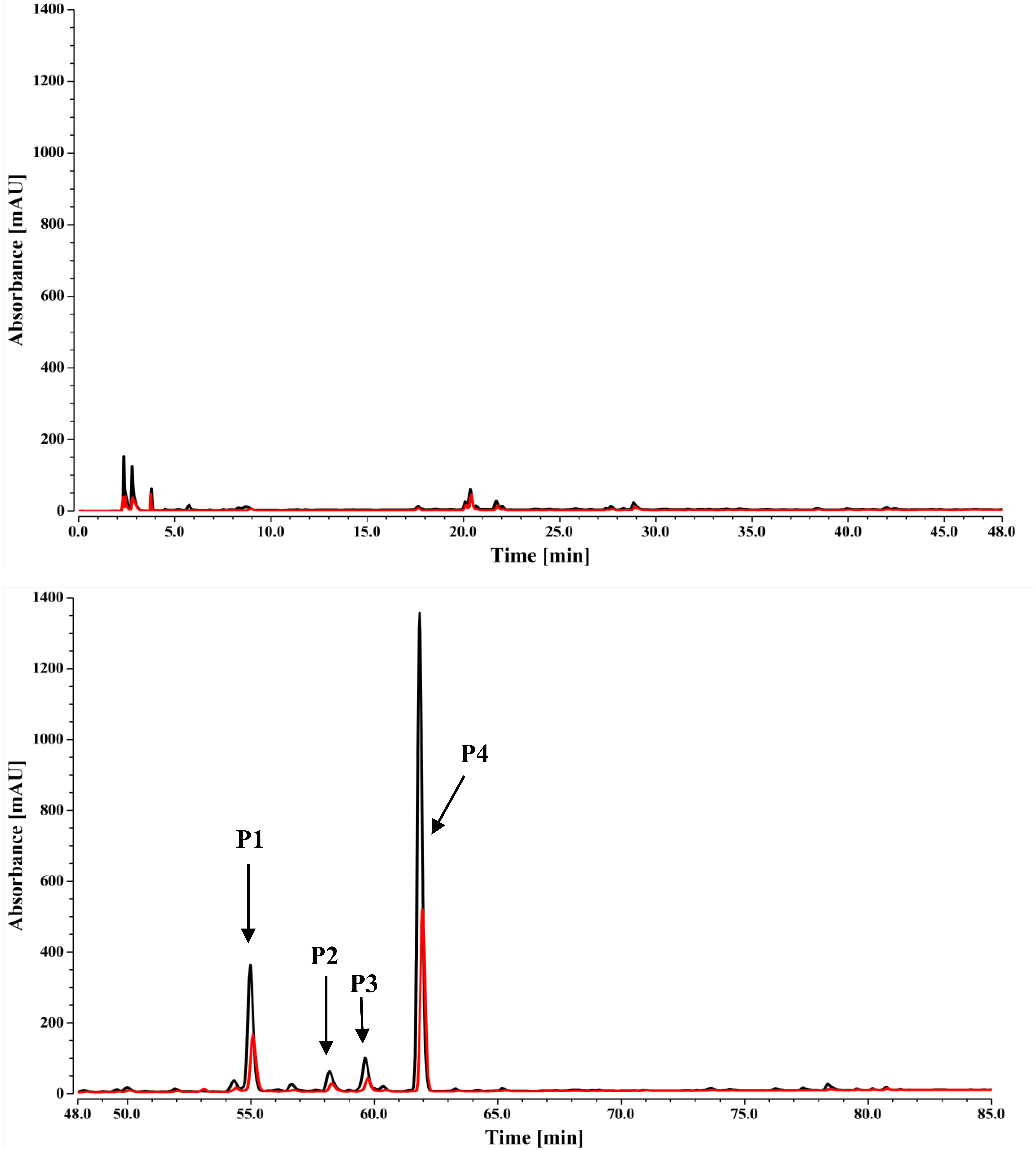

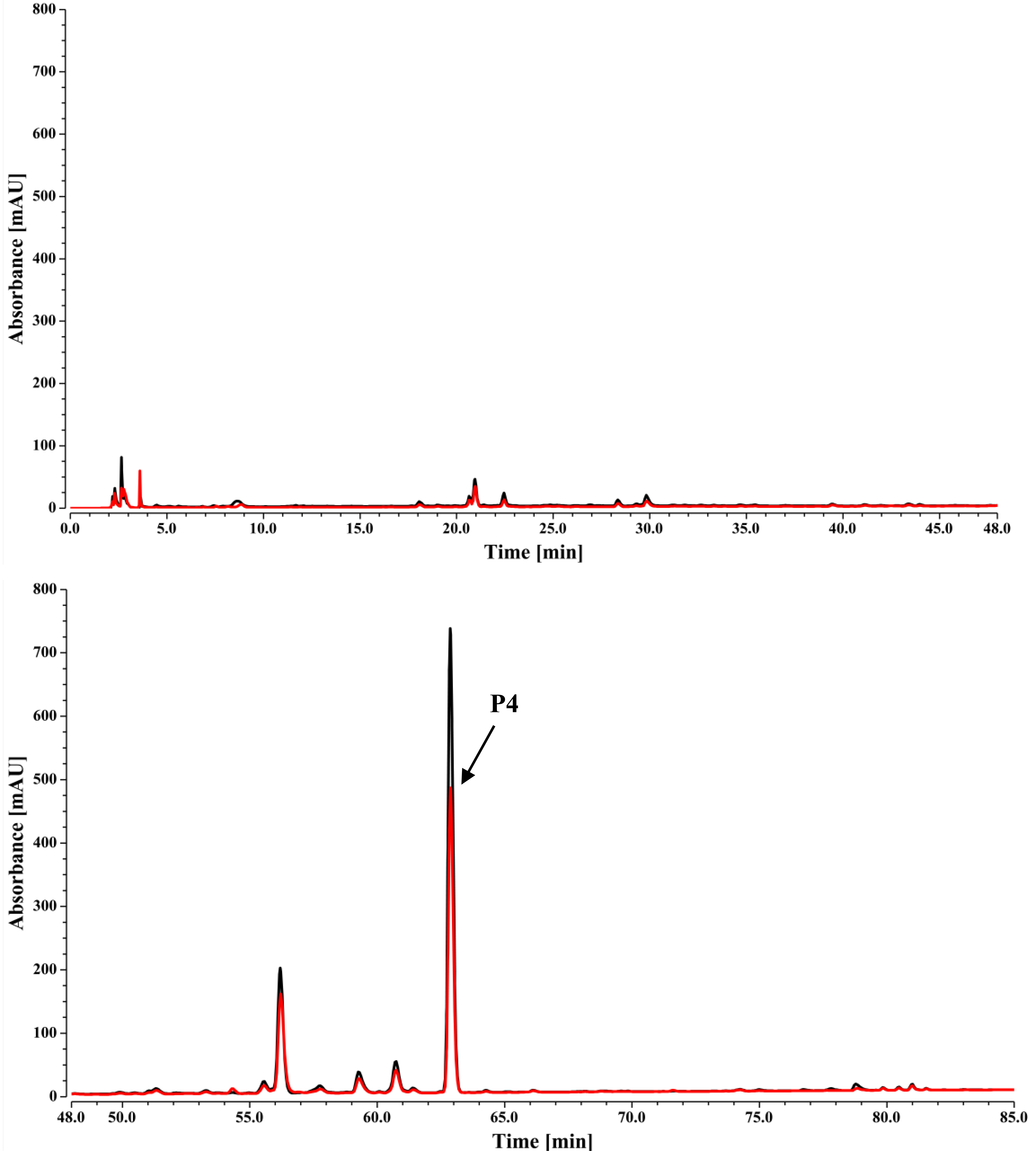


**(B1)**

**(B2)**

**(C1)**

**(C2)**

**Fig. S4.** Effects of incubation time on the screening of bioactive compounds from NRR extract. The incubation times were 60 (A1 and A2; A1, 0–48 min; A2, 48–85 min), 90 (B1 and B2; B1, 0–48 min; B2, 48–85 min) and 120 min (C1 and C2; C1, 0–48 min; C2, 48–85 min). Compared to controls that consisted of denatured hepatic mitochondria (red line), HPLC chromatograms of searched NRR extract showed four peaks (P1–P4) that were enhanced due to specific binding with hepatic mitochondria (black line). The concentration of hepatic mitochondria and NRR sample were 0.50 g/L and 12.38 g/L, respectively.
